# Neurology

**Published:** 1904-09-17

**Authors:** 


(*To be continued.*)

### NEUROLOGY.

Epilepsy.—Two thousand cases of epilepsy have been analysed by Starr[1] with the view of answering the question whether epilepsy is a functional disease or not. We can distinguish several distinct types of Jacksonian epilepsy: (1) there is the motor type, in which a local spasm of one or more parts on one side of the body only—followed by temporary weakness—is the characteristic; (2) the sensory type, in which a sudden hallucinatory perception occurs in any one of the senses followed by a temporary suspension of the power of perception in that sense; (3) the aphasic type, in which a sudden interference in the function of speech takes place, either in its receptive or in its emissive side; (4) the psychical type, in which dreamy states or imperative ideas dominate consciousness, often leading to automatic acts whose object is not clear and of which no conscious memory remains. In all these types the Jacksonian attack is an indication of local irritation of the brain cortex, of some organic disease. Now it is found that no sharp line can be drawn clinically between Jacksonian epilepsy and idiopathic epilepsy. Thus in many cases, 38 per cent., there is an aura, which is in many cases identical in character with the sensation constituting or starting the Jacksonian attack; and it is always uniform in the same patient, just like the Jacksonian attacks. The only difference between a Jacksonian attack of the motor type and an idiopathic attack is the extent of the spasm. It seems probable that whenever we have a general convulsion preceded by a definite form of aura we can conclude that there is a focus of irritation, organic in nature, in a definite locality in the brain. It may be said that in Jacksonian epilepsy there is no loss of consciousness, whereas in idiopathic epilepsy there is such a loss. This difference, however, lies merely in the degree and severity of the cerebral irritation and consequent shock. And the distinction is not absolute, for if a Jacksonian attack becomes very severe consciousness is often lost. Secondly, many cases (39 per cent.) of undoubted maldevelopment of the brain, *i.e.* cases of imbecility, hemiplegia of infancy, diplegia of infancy, are the subjects of epilepsy. The organic disease in such cases may be very apparent, or so slight as to often escape recognition. When cases of epilepsy are carefully studied, it is usually possible to find evidences of some mental or physical sign of degeneracy or of defective evolution, especially when the attacks have begun in infancy or early youth. These facts suggest that just as epilepsy is a symptom of organic disease in the cases of maldevelopment of the brain, it may also be a symptom of organic disease where there is no definite evidence of defect of the brain. Another argument may be drawn from the alleged causes of the affection. The first of these causes is heredity—implying that the patient inherits a weak and probably a defective nervous system — an organically imperfect brain. The same argument can be drawn from many of the other causes, viz. trauma, infectious diseases, sunstroke. From a consideration of these various lines of argument, it seems probable that epilepsy is to be regarded as an organic disease. This is rendered all the more likely in view of the fact that changes, either gross or microscopic, have been found by pathologists in the brains of epileptics. Those found by the older pathologists have not been constant, but recent work has uniformly demonstrated finer microscopic changes in the cells of the cortex of a definite type and character.

Prognosis in Epilepsy.—At a meeting of the New York Academy of Medicine[2] the most diverse views were expressed on the curability of epilepsy. Spratling, of the Craig Colony, said that epilepsy was cured in from 5 to 10 per cent. of all cases, but it required, as a rule, a prolonged course of treatment, never less than two or three years. In support of his statement he gave details of 34 cases which had been cured. Of these cases 20 had begun before the age of four years, 9 between ten and twenty years, whilst the rest were older. Twenty-three had grand mal attacks, 7 grand mal and petit mal attacks, and 3 petit mal only. The average duration of treatment was three years. In some cases there would be no material improvement for a long period, perhaps a year, and then there would be a sudden reduction in the number of seizures. Many of the 34 cases had had the disease for many years, so that even marked chronicity is not a bar to cure. In general the more frequent the attacks on admission the less favourable the prognosis. Half of the cases had a sensory aura. The younger the patient when the epilepsy developed, and the longer the period without proper treatment the more unsatisfactory was the mental condition of the patient. On the other hand, Starr said that in his experience, while one might reduce the number of the attacks, lengthen the interval, make the attacks themselves less severe, and produce general improvement, yet a cure was a very rare thing. Of 250 consecutive cases in private practice he could only recall one patient who had recovered. He thought that the reason for this was that epilepsy was almost always an organic nervous affection, the result of imperfect development of the brain, and not a functional nervous disease. These patients presented stigmata of degeneration, which were not post-epileptic but prior to the disease. His belief that epilepsy had an organic basis did not prevent him from treating the disease, but did prevent him from taking a hopeful view of the results to be expected. Clark said that his own private practice yielded fully 25 per cent. of cases of epilepsy in which the patients had gone two to three years without a seizure. The aura was more elaborate and slower in its development in the more hopeful cases—probably showing that the central nervous system was unwilling to co operate with a certain centre which, as it were, was trying to establish an epileptic seizure. He believed that epilepsy was an organic disease, with demonstrable microscopic changes in the cortex, but nevertheless he could not see why these processes should not be arrested or modified by treatment. Collins believed that 10 per cent. of epileptics could be cured. He was of opinion that epilepsy might or might not be an organic disease. He could not agree with the statement that patients with stigmata of degeneration justified a worse prognosis. A valuable paper on the prognosis in epilepsy has been written by Turner,[3] based on the statistics of the colony at Chalfont St. Peter. Epileptics may be arranged into four classes, with reference to their mental condition in the intraparoxysmal periods—those intellectually normal, those with impaired memory, those who are feeble minded, and lastly the demented. Sex has little influence upon the mental condition. A family predisposition to epilepsy and insanity, though not necessarily militating against the retention of normal mental faculties, favours the supervention of some degree of mental impairment. The duration of the disease influences to some degree the mental condition, mental impairment being less frequent when the fits have lasted under five years than when they have occurred for a longer period. The earlier the onset, especially during infancy and childhood, the less the probability of an unimpaired mental state. The profoundest degrees of dementia are seen when the major and minor attacks co-exist. The petit mal seizures occurring alone are found with the lower grades of mental impairment. When the grand mal occurs alone mental health is as common as mental deficiency. The more frequent the seizures the greater the degree of mental impairment, and *vice versâ*. Fits recurring in series are accompanied by a high grade of dementia. The term "facies epileptica" is applied to the physiognomy characteristic of the disease in many epileptics, *i.e.* an expression of dulness and heaviness with an absence of emotional mobility. This is found more commonly with the higher degrees of dementia, but it is not limited to such cases. The interparoxysmal condition seen in most cases of epilepsy is one expression of the neurosis of which the fits constitute another. The frequency and character-combination of the paroxysms in association with the degree of mental failure indicate the severity and intensity of the disease.

[1] Jour. of Nerv. and Ment. Dis., March. [2] Med. Record, Feb. 6.

[3] Lancet, April 9.

